# Endotoxin as a determinant of asthma and wheeze among rural dwelling children and adolescents: A case–control study

**DOI:** 10.1186/1471-2466-12-56

**Published:** 2012-09-12

**Authors:** Joshua A Lawson, James A Dosman, Donna C Rennie, Jeremy R Beach, Stephen C Newman, Trever Crowe, Ambikaipakan Senthilselvan

**Affiliations:** 1 Canadian Centre for Health and Safety in Agriculture & Department of Medicine, University of Saskatchewan, Saskatoon, SK, Canada; 2 Canadian Centre for Health and Safety in Agriculture, University of Saskatchewan, Saskatoon, SK, Canada; 3 Canadian Centre for Health and Safety in Agriculture, University of Saskatchewan, and the College of Nursing, University of Saskatchewan, Saskatoon, SK, Canada; 4 Department of Medicine, Faculty of Medicine and Dentistry, University of Alberta, Edmonton, AB, Canada; 5 Department of Psychiatry, Faculty of Medicine and Dentistry, University of Alberta, Edmonton, AB, Canada; 6 College of Engineering, University of Saskatchewan, Saskatoon, SK, Canada; 7 Department of Public Health Sciences, School of Public Health Sciences, University of Alberta, Edmonton, AB, Canada; 8 Canadian Centre for Health and Safety in Agriculture Royal University Hospital University of Saskatchewan, 3641-103 Hospital Drive, Saskatoon, SK S7N 0 W8, Canada

**Keywords:** Asthma, Children, Endotoxin, Wheeze, Pediatrics, Allergy

## Abstract

**Background:**

The association between endotoxin exposure and asthma is complex and has been associated with rural living. We examined the relationship between domestic endotoxin and asthma or wheeze among rural school-aged children (6–18 years) and assessed the interaction between endotoxin and other characteristics with these outcomes.

**Methods:**

Between 2005 and 2007 we conducted a case–control study of children 6–18 years in the rural region of Humboldt, Canada. Cases (n = 102) reported doctor-diagnosed asthma or wheeze in the past year. Controls (n = 208) were randomly selected from children without asthma or wheeze. Data were collected to ascertain symptoms, asthma history and indoor environmental exposures (questionnaire), endotoxin (dust collection from the play area floor and child’s mattress), and tobacco smoke exposure (saliva collection). Statistical testing was completed using multiple logistic regression to account for potential confounders and to assess interaction between risk factors. A stratified analysis was also completed to examine the effect of personal history of allergy.

**Results:**

Among children aged 6–12 years, mattress endotoxin concentration (EU/mg) and load (EU/m^2^) were inversely associated with being a case [odds ratio (OR) = 0.44, 95% confidence interval (CI) = 0.20-0.98; and OR = 0.38, 95% CI = 0.20-0.75, respectively]. These associations were not observed in older children or with play area endotoxin.

**Conclusions:**

Our results suggest that endotoxin exposure might be protective for asthma or wheeze. The protective effect is found in younger school-aged, non-allergic children. These results may help explain the inconsistencies in previous studies and suggest that the protective effects of endotoxin in the prevention of atopy and asthma or wheeze are most effective earlier in life.

## Background

Asthma is a chronic respiratory disease that can lead to activity limitation, school and work absenteeism and hospitalization. The environment plays an important role in the development of asthma and in triggering symptom exacerbations although the impact of many environmental exposures remains poorly characterized. One such exposure reported to be associated with asthma or respiratory symptoms is endotoxin. Endotoxin is derived from the cell wall of gram-negative bacteria and is ubiquitous in the environment. It can be pro-inflammatory [1] when encountered as part of a pathogen but can also modify the immune response to potentially protect against atopic disease [2]. Animal models have shown differential allergic responses to endotoxin depending on the timing of exposure [3].

Asthma prevalence has been found to be lower in rural areas [4,5] potentially because of the exposure to farming environments where endotoxin levels may be higher [6]. However, direct studies of household endotoxin exposure and the presence of asthma and wheeze among children have variably described reduced risk [7], increased risk [8] or no association [9,10]. One possible explanation for differences between studies could be interactions between endotoxin and other personal and environmental factors including rural living in associations with asthma.

Previously, we reported on the association between endotoxin and indicators of asthma severity among children with asthma or wheeze [11], the association between endotoxin and diurnal peak flow variability from two week monitoring among children with asthma or wheeze [12], and the association between lung function and endotoxin among children who have asthma or wheeze and children who do not [13]. We sought to expand on this previous research by examining the role of endotoxin on the presence of asthma or wheeze. As such, the purpose of this study was to examine associations between endotoxin exposure and asthma and wheeze in rural-residing children and to investigate the potential effect modification by personal and environmental characteristics on the association between endotoxin exposure and doctor diagnosed asthma or wheeze. We completed this study through the use of a case–control study design from a general population of children and adolescents.

## Methods

### Study design and population

We conducted a case–control study of children living in and around the rural community of Humboldt, Saskatchewan, Canada during the fall and winter seasons from 2005 to 2007 with some catch-up in the spring. All rural schools in the same school district as schools within the community boundary of Humboldt were approached. The total population of the town of Humboldt and its surrounding rural municipality was approximately 6,100 people. Of the towns surrounding Humboldt that had schools included in the study, the population ranged between approximately 200 to 800 people. The major source of industry for the region was classified as agriculture and resources. Subjects were recruited from a previously conducted population-based cross-sectional survey of respiratory health of 6 to 18 year old children which had been completed in 2004. Potential cases comprised all subjects reporting doctor-diagnosed asthma or wheeze in the past 12 months. Two potential controls for each case were randomly selected from among children who were not considered cases. Once selected, an invitation letter was mailed and potential participants were contacted by telephone a maximum of seven times. Following contact, cases and controls status was confirmed by a screening questionnaire that enquired about asthma diagnoses, recent asthma or wheeze events including symptoms, episodes and breathing medication use.

Data collection for this study included an interviewer-administered questionnaire, dust collection from the home for endotoxin, and saliva collection to assess recent tobacco smoke exposure. The Health Research Ethics Board – Panel A (University of Alberta) and the Biomedical Research Ethics Board (University of Saskatchewan) approved the study as did the local school boards. Prior to taking part, parents and children completed consent and assent forms, respectively.

### Questionnaires

The questionnaire, based primarily on previously validated and standardized questionnaires [14-16], and those used in previous respiratory health studies in Saskatchewan subjects [10,17,18] was administered by a trained interviewer to a parent of the subject. Information was collected on respiratory health, socio-demographic factors, general health, family history, birth characteristics, lifestyle, housing characteristics and environmental exposures. Subjects were classified into age groups (≤12 years vs. >12 years) to be comparable with other childhood asthma studies where age ranges were typically between 6–12 years [18,19], and to be able to look at effect modification by age. Season of testing was defined by the date of the home visit and was recorded as spring (March, April, May), fall (September, October, November) and winter (December, January, February) to account for the potential differences in endotoxin and allergy levels by season. Personal history of an allergic condition was determined by presence questionnaire report of hayfever, eczema and respiratory allergies. Respiratory allergies were based on the question “Has this child ever had an allergy (hives, swelling, and/or wheezing) to any of the following: house dust/grain dust/pollen/trees/grasses/mould or mildew/dog/cat/birds or feathers/farm animals?”

### Collection and analysis of dust samples to quantify endotoxin exposure

Samples of household settled dust were collected from the floor of the room where the child spent most of his/her free time (play area); and from the child’s mattress (mattress). Dust was collected following the International Study of Asthma and Allergies in Childhood (ISAAC) protocol [20]. A sock filter made of Connaught satin with a pore size of approximately 5 to 10 μm [21] was used to collect dust. Floors that were mostly carpet had 2 m^2^ vacuumed for 4 minutes. Completely smooth floor (eg. hardwood, laminate, or linoleum) had 4 m^2^ vacuumed for 4 minutes. Dust collection from the bed was completed after all duvets, blankets and sheets that the child slept under were removed as per ISAAC protocol [20]. The length and width of the bed was measured and the whole area of the bed was vacuumed for 2 minutes. Dust was collected by using one of two vacuum cleaners (both were Solaris vacuums made by Miele – S514). Both vacuum cleaners were equipped with a HEPA filter, and temperature and humidity were measured at the time of vacuuming.

According to the ISAAC protocol, vacuums must be capable of at least 800 W of power [20]. For this study, the two vacuums each had a power rating of 900 W. The flow rate vs. static pressure relationships for the vacuums was experimentally tested at the College of Engineering at the University of Saskatchewan. A typical second order polynomial relationship between flow rate and static pressure (R^2^ for all tests exceeded 0.99) was observed in all cases. The performance of the vacuums evaluated post study was found to be comparable to pre-testing evaluation.

Assays to determine endotoxin levels were completed following the protocol by Gereda et al. [22] Laboratory technicians were blinded to the case–control status of the subject and to all other data collected. Quantity of endotoxin was measured using the kinetic chromogenic *Limulus* assay (Cambrex Bio Science, Kinetic QCL, Walkersville, MD). For 49 samples, endotoxin analyses were conducted with the following dilutions: neat, 10, 100 and 10 with a spiked sample. These were all completed in duplicate. The spike recovery was 113%. For the remainder of the samples, analyses were completed in duplicate only using the 10 and 100 dilutions. The coefficient of variation between samples was 9.32%. Endotoxin measures were log-transformed prior to statistical analysis.

Endotoxin levels were expressed as concentration [endotoxin units/mg of dust collected (EU/mg)] and load [endotoxin units/m^2^ of area vacuumed (EU/m^2^)]. Both were reported as there is inconsistency in the literature about which expression is most appropriate to report [23]. While concentration is most often presented, it has been suggested that load may more accurately describe the burden of exposure [24].

### Collection and analysis of saliva samples to quantify cotinine levels

Tobacco smoke exposure was determined by salivary cotinine levels. Subjects were asked to spit into a specimen container without the use of gum, Teflon, or other materials that would stimulate the flow of saliva. Up to 5 ml of saliva were collected. Analysis for cotinine was conducted using saliva cotinine microplate enzyme immunoassay kits (Cozart plc, United Kingdom).

### Statistical analysis

Analysis was completed using STATA version 9.0 (College Station, TX: StataCorp LP). Initially we compared personal and environmental characteristics between cases and controls based on frequencies and proportions. Following this, we assessed the correlation between play area and mattress endotoxin levels using the correlation coefficient with statistical significance adjusted for within family correlation. We also identified the predictors of play area and mattress endotoxin levels using linear regression after log-transforming the endotoxin measures, using generalized estimating equations (GEE) to account for within family correlation, and adjusting for potential confounders. Finally, we assessed the association between the measures of endotoxin and case-status.

In this final set of analyses, the outcome for the multiple logistic regression model was case–control status. A multiple logistic regression model was fitted for each measure of endotoxin that was independent of the other models. Model 1 assessed play area endotoxin concentration; Model 2 assessed play area endotoxin load; Model 3 assessed mattress endotoxin concentration; and Model 4 assessed mattress endotoxin load. Additional variables were included based on statistical significance, clinical importance, and the effect the removal of that variable had on the beta coefficients of other variables in the model. The additional variables included in the Models were: age group, sex, daycare attendance, presence of home air filter, smoking during pregnancy, smooth floors in the bedroom in the first year of life, season of testing, and tobacco smoke exposure. Levels of cotinine (ng/ml) were eventually categorized as high and low post analysis based on the median cotinine level (1.24 ng/ml) due to a highly skewed distribution of results that could not be normalized after log transformation. Other potential confounders, such as parents’ education status, consumption of unpasteurized milk, type of farm exposure (none, grain, livestock), and presence of mold or dampness in the home were tested but not included in the final models as they did not influence the associations of the other variables in the model, and they did not have independent associations with case–control status. Interaction terms of clinical importance were also considered. These included potential interactions between endotoxin and sex, age group, and tobacco smoke exposure. All assessment of interactions was determined a priori. Throughout the analyses, GEE with an exchangeable working correlation were used to account for clustering within families. Occasionally, the model would not converge and an independent working correlation was used. Because of the importance of a personal history of allergy, interaction between endotoxin exposure and the personal history of allergy was assessed and the analysis was repeated after stratification by allergic history.

## Results

Among eligible cases and controls 322 (43.4%) children and their parents agreed to participate in the study. Participation was similar between potential cases and controls (47.0% vs. 41.4%, respectively; p = 0.35). When comparing differences between those who took part in the study and those who did not, using data from the previous cross-sectional study phase, case and control participants had less parental smoking exposure, and, among the controls who participated, were more likely to have an air filter in their home (data not shown). Dust or saliva samples could not be collected for 12 subjects. These subjects were excluded from subsequent analyses resulting in data being available for 102 cases and 208 controls.

The proportion of time living in the current home did not differ by age group or case–control status and was not correlated with any of the endotoxin measures (data not shown). Also, a small proportion of the study population had changed their flooring or changed their bedding because of a family member’s allergic or respiratory condition 12.6% and 14.2%, respectively).

Although the age distribution was similar, personal and family history of allergy was over three times higher in cases than in controls. Cases were also more likely to have used antibiotics in their first year of life, have a mother who smoked during pregnancy, was subject to early respiratory illness, used a home air filter, or participated in haying, mowing or raking lawns in the past year (Table 1). A lower proportion of cases than controls were female, were exposed to pesticides in the past year, had smooth floors in the bedroom in the first year of life, and had testing completed in the winter (Table 1).

**Table 1:** Distribution of characteristics and univariate associations with case–control status

**Characteristics**	**Cases n = 102%**	**Controls n = 208%**	**OR (95% CI)**
>12 years (ref: ≤12 years)	35.3	38.0	0.89 (0.54-1.46)
Female (ref: male)	35.3	60.6	0.38 (0.23-0.60)*
A parent with more than high school education (re: ≤high school)	39.4	37.3	0.91 (0.56-1.49)
Personal history of an allergic condition (ref: No allergic history)	72.5	33.2	4.86 (2.83-8.35)*
Family history of asthma (ref: No family history)	21.6	7.2	3.19 (1.51-6.73)*
Maternal smoking during pregnancy (ref: No smoking in pregnancy)	20.6	9.1	2.64 (1.36-5.11)*
Used antibiotics in 1^st^ year (ref: None in the 1^st^ year)	56.9	37.0	2.07 (1.28-3.35)*
Early respiratory illness (ref: No early respiratory illness)	18.6	8.7	2.36 (1.09-5.11)*
Presence of mold or dampness in the home (ref: none)	55.8	57.8	1.09 (0.67-1.76)
Home air filter present (ref: No air filter)	10.8	3.8	3.13 (1.14-8.62)*
Home humidifier present (ref: No humidifier)	30.4	21.6	1.52 (0.85-2.63) ^†^
Indoor burning sources present (ref: No indoor burning)	32.4	41.3	0.58 (0.34-1.01) ^†^
Currently with a pet (ref: No current pet)	77.5	70.2	1.32 (0.74-2.37)
Any pesticide exposure (ref: No pesticide exposure)	62.7	75.5	0.52 (0.29-0.90)*
Haying in the past year (ref: Did not hay in past year)	29.4	17.8	2.01 (1.10-3.65)*
Mowed or raked in the past year (ref: Did not mow/rake in past year)	83.3	72.6	2.00 (1.08-3.72)*
Ever lived on a farm or visited a farm regularly (ref: Did not live on a farm or visit a farm regularly)	60.8	66.8	0.79 (0.47-1.33)
Smooth floors in the bed room in the first year (ref: No smooth floors in first year)	5.9	13.9	0.25 (0.07-0.88)*
Season of testing			
Spring (Reference)	66.7	48.1	1.00
Fall	22.5	22.6	0.79 (0.41-1.53)
Winter	10.8	29.3	0.28 (0.13-0.57)*
High tobacco smoke exposure (ref: low exposure)	48.0	51.0	0.99 (0.64-1.54)

Statistical comparisons were carried out by logistic regression using generalized estimating equations to account for clustering within families; OR = odds ratio, CI = confidence interval.

* p < 0.05; ^†^ p < 0.10.

Play area endotoxin concentration was correlated with mattress endotoxin concentration (r = 0.42, p < 0.001). Similarly, Play area endotoxin load was correlated with mattress endotoxin load (r = 0.30, p < 0.001). Higher play area endotoxin levels were associated with the presence of mice and a family history of allergy while lower play area endotoxin levels were associated with higher education of the parent (Table 2). Higher mattress endotoxin levels were associated with the presence of mice, cats, ETS, home crowding, and winter season while lower mattress endotoxin levels were associated with air conditioning, farm or acreage dwelling, and older age (Table 2). Finally, there was no association between personal history of allergy and any measures of endotoxin including play area endotoxin concentration [odds ratio (OR) = 1.15, 95% CI = 0.66-2.00], play area endotoxin load (OR = 0.98, 95% CI = 0.65-1.48), mattress endotoxin concentration (OR = 1.61, 95% CI = 0.95-2.73), and mattress endotoxin load (OR = 0.91, 95% CI = 0.59-1.43).

**Table 2:** Determinants of play area and mattress endotoxin levels

	**Play area endotoxin concentration (EU/mg) β (SE)**	**Play area endotoxin load (EU/m**^**2**^**) β (SE)**	**Mattress endotoxin concentration (EU/mg) β (SE)**	**Mattress endotoxin load (EU/m**^**2**^**) β (SE)**
Age >12 years (ref: <12)	0.02 (0.04)	0.08 (0.06)	−0.12 (0.05) *	−0.11 (0.06) *
Parent with > high school (ref: ≤high school)	−0.10 (0.05) †	−0.17 (0.08) *	−0.03 (0.05)	−0.003 (0.07)
Family history allergy (ref: no family history)	0.13 (0.06) *	0.21 (0.09) *	0.09 (0.05) *	0.09 (0.06)
ETS exposure (ref: none)	−0.01 (0.08)	−0.03 (0.11)	0.10 (0.05) *	0.10 (0.07)
Rural home (ref: non-rural)	−0.02 (0.13)	0.07 (0.15)	−0.25 (0.14) †	−0.33 (0.16) *
Mice present (ref: none)	0.24 (0.09) *	0.34 (0.11) *	0.13 (0.07) †	0.23 (0.10) *
Home air conditioning (ref: none)	−0.11 (0.06) †	−0.04 (0.08)	−0.05 (0.04)	−0.15 (0.06) *
Fall (ref: spring)	−0.12 (0.11)	−0.04 (0.13)	−0.06 (0.06)	0.09 (0.08)
Winter (ref: spring)	−0.10 (0.10)	−0.002 (0.13)	0.05 (0.06)	0.22 (0.07) *
Cat present in past 12 months (ref: none)	0.14 (0.10)	0.11 (0.13)	0.18 (0.08) *	0.23 (0.11)
Crowding	−0.07 (0.08)	−0.03 (0.11)	0.25 (0.09) *	0.21 (0.12) †

Adjusted for each of the variables in the table as well as sex, haying, graining, grooming, living on a farm, ants, age of home, gas heating, air conditioning, current pets, dogs, season of testing, fireplace, ETS exposure, hours of open windows, changing of smoking habits because of a child’s condition, TV watching and case–control status. Endotoxin levels are log transformed.

* p < 0.05; † p < 0.10.

**Figure 1:**
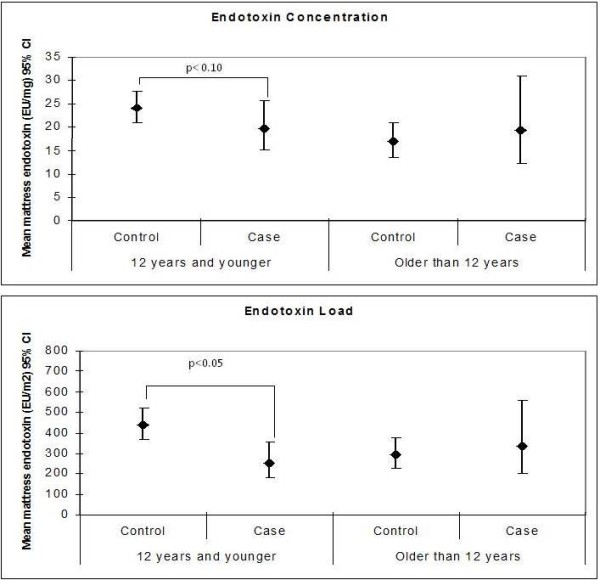
Geometric mean mattress endotoxin concentration and load (95% CI) by case–control status and age group *. * Statistical comparisons between cases and controls were completed within age group strata using general linear models with GEE to account for clustering within families; Endotoxin levels were log transformed prior to the analysis.

Controls had significantly higher mean (geometric) mattress endotoxin loads compared to cases (Table 3). The differences in endotoxin concentration (EU/mg) and load (EU/m^2^) from the play area and for mattress endotoxin concentration were not statistically significant. When comparing levels of mattress endotoxin by case–control status after stratification by age group there was no difference in endotoxin levels (concentration or load) by case–control status (Figure 1) among children older than 12 years. However, among children aged 12 years and younger, controls had higher mattress endotoxin load (p = 0.002) than cases with a similar trend seen for mattress endotoxin concentration, although this was not statistically significant (p = 0.10; Figure 1).

**Table 3:** Distribution of endotoxin load and concentration among cases and controls by source of dust sample

	**Controls N = 208 Geometric mean (95% CI)**	**Cases N = 102 Geometric mean (95% CI)**
Play area endotoxin		
Concentration (EU/mg)	40.8 (35.7-46.6)	51.8 (42.0-63.9)
Load (EU/m^2^)	817.3 (674.8-989.8)	868.2 (672.9-1119.9)
Mattress endotoxin		
Concentration (EU/mg)	21.1 (15.6-24.8)	19.6 (15.6-24.8)
Load (EU/m^2^)	376.2 (324.4-436.2)	240.5 (212.1-370.9) *

Statistical comparisons were completed by general linear model using generalized estimating equations to account for clustering within families; Endotoxin levels were log transformed prior to analysis.

* p < 0.05 between cases and controls.

The associations between play area endotoxin concentration or load and case–control status were not statistically significant after adjusting for potential confounders [odds ratio (OR) = 1.64, 95% CI = 0.73-3.70 for endotoxin concentration and OR = 1.10, 95% CI = 0.64- for endotoxin load]. There was a statistically significant interaction between mattress endotoxin load and age group (p = 0.02). While the interaction between mattress endotoxin concentration and age group followed similar trends, it was not statistically significant (p = 0.12). Mattress endotoxin was inversely associated with being a case among those who were 12 years or younger [odds ratio (OR) = 0.44, 95% CI = 0.20-0.98 for endotoxin concentration and OR = 0.38, 95% CI = 0.20-0.75 for endotoxin load; Table 4]. All of the models appeared to have a good fit based on the Hosmer-Lemeshow test where no model showed statistical significance (p > 0.05) and inspection of model diagnostics suggested a good model fit.

**Table 4:** Results from multiple logistic regression analyses* for case–control status with mattress endotoxin among the total population and by personal history of an allergic condition

	**Total population**		**Without a personal history of an allergic condition**		**With a personal history of an allergic condition**	
	**n**	**OR (95% CI)**	**n**	**OR (95% CI)**	**n**	**OR (95% CI)**
Mattress endotoxin concentration (EU/mg)						
Among those aged ≤12 years	195	0.44 (0.20-0.98) ^†^	132	0.20 (0.06-0.68) ^†^	63	0.45 (0.16-1.29)
Among those aged >12 years	115	1.18 (0.42-3.31)	83	0.66 (0.11-3.67)	32	3.36 (0.53-21.23)
Mattress endotoxin load (EU/m^2^)						
Among those aged ≤12 years	195	0.38 (0.20-0.75) ^†^	132	0.22 (0.08-0.62) ^†^	63	0.50 (0.20-1.28)
Among those aged >12 years	115	1.31 (0.47-3.68)	83	0.46 (0.09-2.42)	32	4.02 (0.99-16.41) ^‡^

* Each model is fitted with one measure of endotoxin and is independent of the other models; Each model is adjusted for age group, sex, daycare attendance, presence of home air filter, smoking during pregnancy, smooth floors in the bedroom in the first year of life, season of testing, tobacco smoke exposure, and age group*endotoxin; Endotoxin levels were log transformed prior to analysis; OR = Odds ratio; CI = Confidence interval; EU = Endotoxin units.

^†^ p < 0.05; ^‡^ p < 0.10.

p = 0.002.

p = 0.10.

Because of the important influence of personal allergy in the relationships with endotoxin, we assessed the interaction between endotoxin exposure and personal history of allergy in the relationship with asthma and wheeze. There was no statistically significant interaction between personal history of allergy and floor endotoxin for either concentration (= − 0.22) or load (p = 0.20). However, there was a statistically significant interaction between mattress endotoxin concentration (p = 0.043) and mattress endotoxin load (p = 0.049). Because of this, we stratified our analysis for mattress endotoxin by personal history of allergy. Among those without a personal history of allergy, there were statistically significant inverse associations between mattress endotoxin (concentration and load) and case–control status among children 12 years and younger (Table 4). Among children 12 years and older with a personal history of allergy, there was a trend towards an increased risk of being a case associated with mattress endotoxin load (Table 4).

## Discussion

Our results demonstrate the complex nature of the association between endotoxin and asthma in children. Higher mattress endotoxin was associated with a reduced risk of asthma/wheeze but the effect was greatest among children 12 years and younger and without a personal history of allergic disease. In contrast, among children 12 years and older with a personal history of allergic disease, there was a suggestion of increased risk of asthma or wheeze with higher mattress endotoxin load. While an association between asthma and related outcomes with endotoxin has been reported previously, we add to the previous results by showing that these associations may differ depending on various personal characteristics. We also suggest that the source of dust is important to consider. These findings may help explain some of the inconsistency between previous results and help explain endotoxin’s role in asthma and wheeze.

In a study from Germany, Austria and Switzerland, inverse associations between endotoxin load (EU/m^2^) from mattress dust and atopic asthma and wheeze were observed in children (6 to 13 years) [7]. A case–control study from England that included 4–17 year olds showed that endotoxin concentration from the living room floor dust was associated with an increased risk of asthma [8]. A case–control study from Saskatchewan that included 6–12 year olds did not find an association between either play area floor endotoxin or mattress endotoxin although it did find an association between endotoxin and school absenteeism among those cases with allergy [10]. Despite the differences in results from the other two case control studies [8,10] and our study, we expand on the previous studies by showing that the inconsistencies in the results may depend on other characteristics such as age and personal history of allergy.

Similarly, results from other studies have found that the association between endotoxin and asthma may be related to allergy. Despite showing a protective effect of endotoxin on atopic asthma or wheeze, the study from Germany, Switzerland and Austria also found an increased risk of non-atopic wheeze with higher endotoxin levels among children from non-farming households [7] showing some disagreement with ours. A separate case–control study in Palestine that included 6 to 12 year old children [25] showed inverse associations between medium endotoxin levels from the mattress and being a non-sensitized case (report of wheeze in the past 12 months) compared to a non-sensitized control [25], in agreement with the results of our study. We found stronger protective effects of endotoxin among those without a history of allergic disease. However, our observations regarding the personal history of allergy should be interpreted with some caution because of the lack of an objective measure of allergy and may account for the differences between our study and the study by Braun-Fahrländer et al. [7,25] One explanation for our results could be the timing and duration of endotoxin exposure, both factors which could affect allergy and asthma onset. It may be that among those who did not have a history of allergy there was more early exposure to endotoxin prior to allergen exposure while those with a history of allergy had earlier exposure to allergens leading to sensitization. With continued exposure to endotoxin, the asthma response would be prevented among the non-sensitized but potentially increased in the sensitized individuals. This is somewhat supported by the strong protective effect in younger, non-allergic children but a weaker and non-significant association in younger, allergic children and with a trend towards increased risk of asthma associated with endotoxin in older, allergic children seen in our study. This hypothesis is supported by results by Tulic et al. showing that the timing of endotoxin exposure relative to sensitization could prevent or increase allergic and asthma responses in animals. A related explanation may stem from Radon’s outline where high allergen exposure and low endotoxin exposure could result in a higher prevalence of atopic asthma. Children in this community may experience similar levels of allergens regardless of allergy status. As such, those with allergic disease may be more likely to develop asthma with the given allergen levels and without the protective effect of endotoxin.

Studies involving infants have shown increased risk of wheeze associated with higher endotoxin levels [26]. Despite this, a change in the association with increasing age has also been reported [27]. Among preschool children (<5 years), there was an increased risk of wheeze with higher endotoxin levels measured at baseline but this association became reduced as follow-up increased from 22 months to 46 months, resulting in an inverse association by the time the children reached ages 5 to 9 years [27]. However, this was a highly selected population of siblings in a birth cohort of children with a parental history of allergic disease. Our study further suggested either no association or a positive association among adolescents. A national study from the US that included children, adolescents and adults showed that increased endotoxin from the bedroom floor or mattress was associated with increased risk for various indicators of asthma and wheeze but only among adults [9]. These findings support the contention that the association between asthma and endotoxin may be age-dependent.

The inverse associations with the presence of asthma and wheeze may reflect long-term effects resulting from more complex immuno-modulatory processes. Exposure to endotoxin is thought to initiate a cascade of events via CD14 and TLR4 that results in the release of mediators shifting the immune response towards a Th1 response and away from the atopic Th2 response [28]. These pathways may be modified by a number of factors identified through animal models including the timing of exposure, the dose of the exposure, and the frequency of exposure, resulting in the reported differences in the association between endotoxin and respiratory outcomes such as asthma and wheeze [3,29,30]. Another consideration that may influence the associations is the possibility of gene-environment interactions. Previous studies have shown that depending on the CD14 polymorphism, the association between asthma and endotoxin can vary and include being a protective factor as well as a risk factor [31,32].

We should also consider the possibility that these associations result not from the effect of endotoxin on asthma, but the effects of having asthma or wheeze on the remediation of endotoxin in homes. If, for example, the parents of young children were more scrupulous about vacuuming their child’s mattress if they had asthma, this might explain the association seen. Such an association might also be postulated to become less strong as children aged, and parental influence on the bedroom environment diminished. When we considered the results after adjusting for changes that were made in the home in response to a family member’s allergic or respiratory condition, the results were similar (data not shown). Unfortunately, other than consideration of these variables, it is not possible within these data to know if the temporal sequence of exposure and disease, but it is apparent that a similar effect was not seen for play area endotoxin that would likely be subject to the same parental cleaning effects for children under 12.

It has been suggested that the possible protective effects of endotoxin on the presence of asthma results from early life exposures to endotoxin. While we were unable to assess this early life association directly, our study population was relatively stable and a low proportion of families had changed homes, flooring or bedding because of an allergic or respiratory condition of a family member.

Studies have reported that the determinants of endotoxin differed between locations sampled in the home [10,33] suggesting that the endotoxin types could differ between sampling locations. A study that has characterized the types of domestic endotoxin by the length of fatty acid chain has shown that there are qualitative differences between sampling locations in the home [34]. Researchers from China reported differences in the health effects of endotoxin depending on the length of fatty acid chain of the endotoxin collected from schools [35]. These observations, along with ours, may help to explain some of the inconsistencies in reported associations and suggest the use of multiple sampling locations or characterization of endotoxin in future studies.

When comparing levels of endotoxin measured in our study, we were in the low to normal range of other studies when considering endotoxin concentration (EU/mg) but much lower than other studies when considering endotoxin load (EU/m^2^) [9,10]. This suggests that there may be more endotoxin attached to the dust collected from our study population but a lower overall burden of endotoxin in the homes, possibly due to less dust or smaller size of dust particles than in other study populations. This difference in levels of endotoxin compared to other study populations emphasizes the importance of considering endotoxin expression by both concentration and load especially given that there is not a consistent use of expression type.

Several limitations of our study should be considered. While we incorporated a case–control design, data collection for this analysis was at one point in time and used prevalent cases making it vulnerable to cross-sectional limitations including a lack of temporal assessment. Due to practical considerations, this is typical of asthma case–control studies [8,10]. Also, no objective measures of allergen exposure such as pet allergen or molds, or other airborne contaminants such as beta glucans were collected. It may be that cases were exposed to a higher level of allergens than were controls. While there was not a statistically significant difference in the exposure to pets or report of mold between cases and controls, there still could be differences in the allergen levels between the two groups. Strengths of our study included the use of objective measures of exposure assessment for the primary exposures (endotoxin and tobacco smoke) thus limiting the likelihood of recall bias in these associations. In addition, the instruments used were based on standardized questionnaires and the research nurses were carefully trained and supervised while laboratory technicians were blinded.

## Conclusions

In conclusion, endotoxin appears to be associated with a decreased likelihood of having asthma or wheeze among children 12 years and younger and whose parents did not report a personal history of allergic disease. We also found that the source of the domestic dust sample is an important consideration when investigating associations between endotoxin and asthma. Finally, the results for endotoxin load were typically stronger and more consistent than for endotoxin concentration suggesting that load more be a more relevant measure of endotoxin exposure when related to human health. These results suggest that the effects of endotoxin may be different for certain subpopulations and that multiple sampling sites or characterization of endotoxin in future studies should be considered to help elucidate the role of endotoxin in the development of asthma and explain the inconsistencies observed between studies.

## Abbreviations

CD14: Cluster of differentiation 14; CI: Confidence interval; EU: Endotoxin unit; GEE: Generalized Estimating Equations; ISAAC: International Study of Allergy and Asthma in Children; m: Meter; mg: milligram; ml: millilitre; ng: nanogram; OR: Odds ratio; p: p-value; Th1: T-helper 1; Th2: T-helper 2; TLR4: Toll like receptor 4; vs: Versus.

## Competing interests

The author(s) declare that they have no competing interests.

## Authors’ contributions

All of the authors participated in the conception and design of the study; the planning of data collection; the interpretation of results; revising and approval of the manuscript. JL coordinated all phases of this project and drafted the initial manuscript.

## Funding

Canadian Institutes of Health Research (Grant no: MOP-57907).

## Pre-publication history

The pre-publication history for this paper can be accessed here:

http://www.biomedcentral.com/1471-2466/12/56/prepub
